# Prevalence of the *Cryptosporidium* Pig Genotype II in Pigs from the Yangtze River Delta, China

**DOI:** 10.1371/journal.pone.0020738

**Published:** 2011-06-03

**Authors:** Jianhai Yin, Yujuan Shen, Zhongying Yuan, Weiyuan Lu, Yuxin Xu, Jianping Cao

**Affiliations:** National Institute of Parasitic Diseases, Chinese Center for Disease Control and Prevention, Key Laboratory of Parasite and Vector Biology, Ministry of Health, WHO Collaborating Centre for Malaria, Schistosomiasis and Filariasis, Shanghai, People's Republic of China; University of South Alabama, United States of America

## Abstract

**Background:**

*Cryptosporidium* spp. is prevalent globally, pigs are an important *Cryptosporidium* reservoir. In China, little data regarding rates of *Cryptosporidium* infections in pigs are available. The present study was therefore aimed at characterizing the distribution of *Cryptosporidium* species in pigs from two different cities, Shaoxing and Shanghai, from the Yangtze River delta.

**Methodology/Principal Findings:**

Nested PCR to amplify the 18S rRNA locus on DNA extracted from fecal samples (n = 94) revealed the positive rate of *Cryptosporidium* in pigs from two cities was approximately 17.0%. The positive rates in Shanghai and Shaoxing were 14.3% and 25.0% respectively. Amplified sequences were verified by sequencing. The identified strain belonged to the *C.* pig genotype II using BLAST analysis in the NCBI database.

**Conclusion/Significance:**

Our finding of *Cryptosporidium* pig genotype II in pigs in the Yangtze River delta area suggests that pig farms in this region must be considered a public health threat and proper control measures be introduced.

## Introduction

*Cryptosporidium* is a protozoan parasite that can cause severe diarrhea, anorexia and weight loss, especially in neonatal and immunocompromised animals. To date, more than 20 *Cryptosporidium* species have been identified [1], [2], however, more than 60 genotypes remain undefined [1], [2], [3]. *Cryptosporidium* species can parasitize a wide spectrum of animals and are the cause of zoonotic infections affecting humans and infections are typically caused by several species (and genotypes) including *C. parvum*, *C. hominis*, *C. canis*, *C. felis*, *C. cuniculus* and *C. meleagridis*. Less frequently associated with human infections are strains including *C. suis*, *C. muris*, *C. ubiquitum* and the rare *Cryptosporidium* pig genotype II [4], [5], [6], [7]. Pigs are an important *Cryptosporidium* reservoir making it important to understand the prevalence of *Cryptosporidium* species in swine as a means of controlling and preventing cryptosporidiosis in both animals and humans. In China, however, little data regarding rates of *Cryptosporidium* infections in pigs are available. The present study was therefore aimed at characterizing the distribution of *Cryptosporidium* species in pigs from two different cities, Shaoxing and Shanghai, from the Yangtze River delta.

## Materials and Methods

### Sampling

In total, 94 fecal samples (50 g) were collected randomly from different animals immediately after defecation into individually labelled plastic bags with a gloved hand from 6 pig farms in Shanghai and 1 from Shaoxing between April 2009 and October 2009.

### Stool Sample Processing

Approximately 20 g of each sample was suspended in 50 ml of deionized water, vortexted and filtered through the sieve. Filtrates were poured back into the same centrifuge tube and centrifuged at 3000 rpm for 10 min. Pellets were then resuspended in 15 ml fresh deionized water and 1.5 ml were transferred into a 2 ml microcentrifuge tube and centrifuged at 3000 rpm for 10 min. Supernatants were discarded and pellets stored at −70°C until use.

### DNA Extraction and PCR Amplification

Genomic DNA was extracted using the QIAamp DNA Stool Mini Kit (Qiagen, Valencia, CA). Supernatants containing DNA were stored at −20°C until use. Nested PCR was used to amplify an approximately 840 base pair (bp) long fragment corresponding to the *Cryptosporidium* 18S rRNA gene using two sets of oligonucleotide primers: TTCTAGAGCTAATACATGCG and CCCATTTCCTTCGAAACAGGA for primary PCR and GGAAGGGTTGTATTTATTAGATAAAG and CTCATAAGGTGCTGAAGGAGTA for secondary PCR [8], [9], [10]. The PCR reactions were carried out in 25 µl volumes containing 12.5 µl 2× Taq Green Master Mix (Promega, Madison, WI), 1 µl of each primer (10 µM), 9.5 µl nuclease-free water (Promega) and 1 µl DNA template. For amplification, templates were subjected to a hot start at 94°C for 1 min followed by 35 cycles of 94°C for 10 s, 55°C for 30 s and 72°C for 1 min followed by a final extension at 72°C for 10 min. Secondary PCR products were analyzed by 2% agarose gel electrophoresis and ethidium bromide staining.

### DNA Sequencing and Analysis

Positive secondary PCR products were subjected to two directional sequencing with secondary primers by the Shanghai Biotechnology Co. Ltd. (Shanghai, China). Amplified sequences were blasted against sequences in the NCBI database and then deposited in GenBank. *Cryptosporidium* isolates identified were compared phylogenetically using the MEGA 4.1 software.

## Results

**Figure 1:**
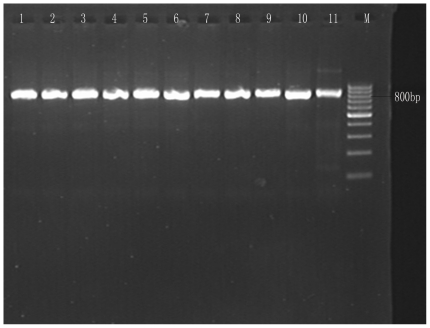
Agarose gel electrophoresis of Shanghai sequences. PCR amplification products of respective *Cryptosporidium* isolates were subjected to 2% agarose gel electrophoresis. Lane 1, positive control, lanes 2–11 18S rRNA amplification products and M, 100 bp DNA ladder. PCR products are approximately 840 bp.

**Figure 2:**
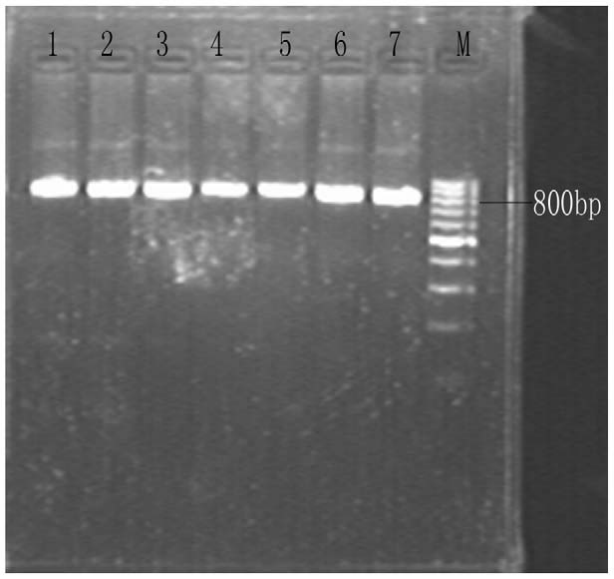
Agarose gel electrophoresis of Shaoxing sequences. PCR amplification products of respective *Cryptosporidium* isolates were subjected to 2% agarose gel electrophoresis. Lanes 1–6 represent secondary 18S rRNA amplification products, lane 7 positive control and M, 100 bp DNA ladder. PCR products are approximately 840 bp.

**Figure 3:**
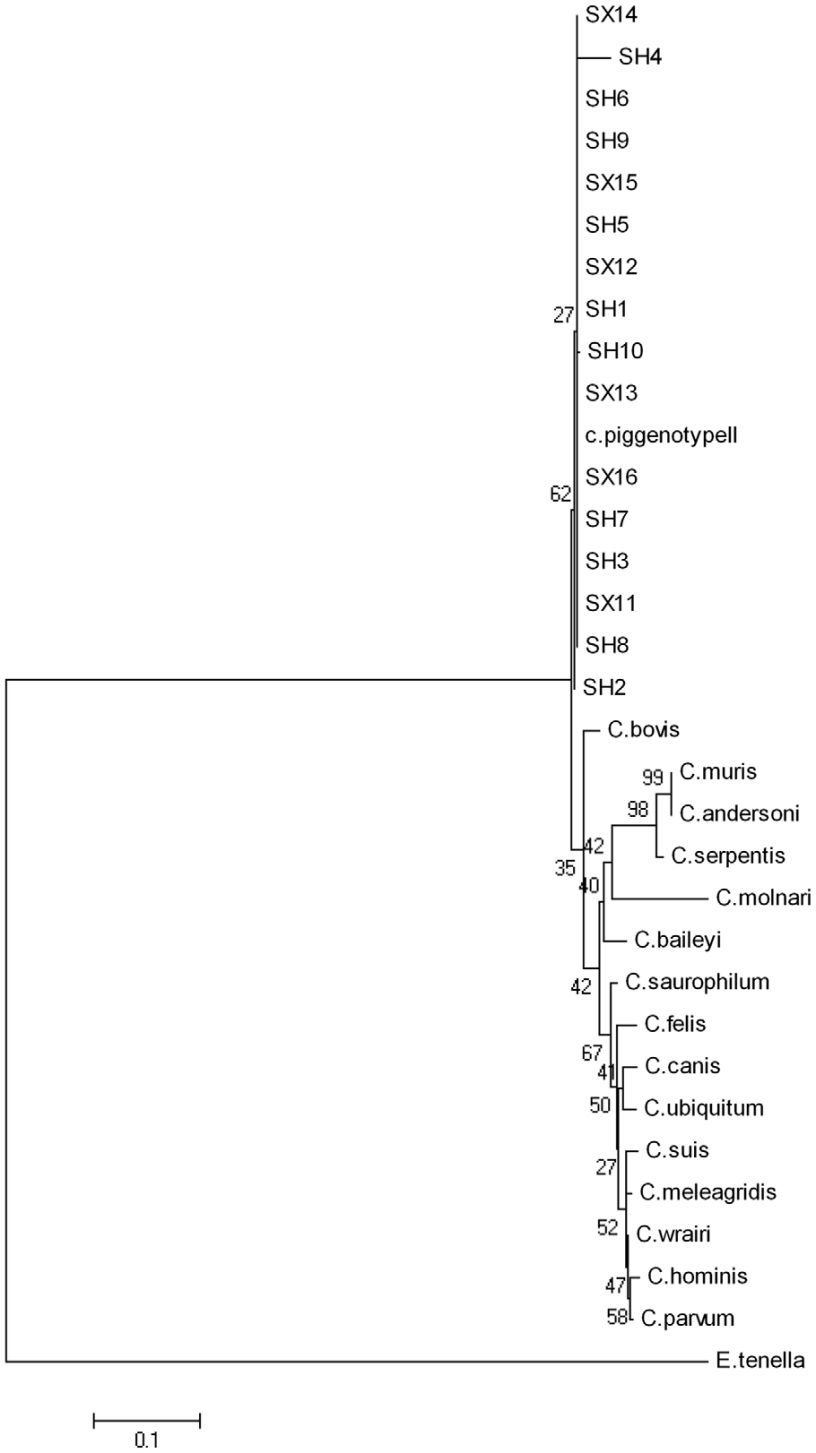
Phylogenetic analysis. 18S rRNA sequences from the respective *Cryptosporidium* isolates identified were compared phylogenetically using the MEGA 4.1 software. The phylogenetic tree obtained demonstrates the genetic diversity of the *Cryptosporidium* genotype II isolates identified from stool samples from Shanghai (SH1–10) and Shaoxing (SX11–16). Analysis was based on the nested-PCR of the18S rRNA locus sequence using the tree method of Neighbor Joining.

Fecal samples (n = 94) were collected from swine farms (24 samples from Shaoxing and 70 from Shanghai). PCR amplification of the 18S rRNA gene locus identified 16/94 (17.0%) *Cryptosporidium* species with 14.3% (10/70) in Shanghai (Figure 1) and 25.0% (6/24) in Shaoxing (Figure 2). BLAST analysis of positive secondary PCR products revealed that all sequences belonged to the *Cryptosporidium* pig genotype II. Phylogenetic relationships between *Cryptosporidium* sequences identified from samples collected from Shanghai and Shaoxing (SH1–10 and SX11–16) and the *Cryptosporidium* pig genotype II (GenBank: GU254170.1) demonstrated in Figure 3.

The nucleotide sequences of *Cryptosporidium* pig genotype II from pigs in this study deposited in GenBank under accession numbers HQ844719–HQ844728 (SH1–SH10) and HQ844729–HQ844734 (SX11–SX16).

## Discussion

*Cryptosporidium* is shed in feces as oocysts that can be transmitted to humans or animals by contaminated water or food. Pigs have been recognized as important reservoirs for several *Cryptosporidium* species/genotypes, some of which are zoonotic pathogens. In the present study, all of the positive samples belonged to the *Crytosporidium* pig genotype II following BLAST analysis against NCBI sequences separately and the phylogenetic analysis..

In Denmark, Langkjaer *et al.* (2007) genotyped *Cryptosporidium* species at the 18S rDNA and/or the *hsp* 70 gene. This analysis identified *Cryptosporidium suis* and/or the *Cryptosporidium* pig genotype II in 183 pigs, in addition to demonstrating an age-specific distribution of species/genotypes. In contrast to the present study the Danish study identified infections with more than 1 species/genotype [11]. In Western Australia, Johnson *et al.* (2008) identified in pre- and post-weaned pigs bred indoors and outdoors a 21.1% prevalence of *Cryptosporidium suis* and *Cryptosporidium* genotype II infections using a nested PCR based 18S rRNA amplification approach, however, the Australian study did not identify age-dependent associations with respect to infection status [12]. In the Czech Republic, Kvac *et al.* (2009) reported that the *Cryptosporidium* infection prevalence in slaughtered finisher pigs and sows was 29% and 10%, respectively, using microscopic analysis. In finishers, mixed *C. suis* and the *Cryptosporidium* pig genotype II infections were detected and *C. parvum* infections in sows were identified [13]. Another study by the same group also showed the age-related prevalence of *Cryptosporidium suis*, *C. muris* and *Cryptosporidium* pig genotype II [14]. These three species and genotypes were also identified in pig slurry samples from an Irish study [15].

In China, studies carried out in different provinces demonstrated using morphologic analysis that 1) *C. parvum* infections were detected in 58/577 pigs in Anhui province with differences in infection rates dependent on age but not sex [16], 2) *C. suis* infections in Chongqing province were identified in 14 pigsties independent of age [17], 3) in Eastern China, including Jiangsu province and Shanghai, *C. parvum*-like genotype infections were identified in pigs using reverse transcription PCR amplification of SSU rRNA [18] and 4) in the present study, only the *Cryptosporidium* pig genotype II was identified in the two districts examined. Additional studies will be needed to identify additional geographic areas where these infections may be present to fully define the nature of *Cryptosporidium* species infections in pigs.

Several *Cryptosporidium* species/genotypes isolated from pigs in China, including *C. suis*, *C. muris*, *C. parvum* and the *Cryptosporidum* pig genotype II have been associated with zoonotic infections. In this study, we identified for the first time the presence of the *Cryptosporidium* pig genotype II in the Yangtez River delta suggesting that prevalence of *Cryptosporidium* infections in swine populations must be carefully monitored since pigs are an important *Cryptosporidium* reservoir that could facilitate zoonotic infections affecting human populations.
